# How to extend your (polylactosamine) antennae

**DOI:** 10.1016/j.jbc.2020.100212

**Published:** 2021-01-14

**Authors:** Matthew S. Kimber

**Affiliations:** Department of Molecular and Cellular Biology, University of Guelph, Guelph, Ontario, Canada

**Keywords:** B3GNT, β1-3-N-acetylgucosaminyltransferase, B4GAL, β-1-4-galactosyltransferase, GT, glycosyltransferase

## Abstract

The elongated antennae decorating eukaryotic glycans are built from polylactosamine repeats. Polylactosamine forms a lectin recognition site and also acts as a platform for presenting diverse additional modifications (*e.g.*, terminal cell-surface antigens); it therefore plays important roles in cell adherence, development, and immunity. Two new papers present a detailed structural and mechanistic investigation of β1-3-N-acetylgucosaminyltransferase 2, a key enzyme in antennae synthesis. The resulting insights will also help decipher other members of GT31, the single largest human glycosyltransferase family.

The disaccharide repeat [-3Galβ1-4GlcNAcβ1-]_n_ is an extremely common recurring motif in eukaryotic glycans. Termed poly-N-acetyl-lactosamine, or polylactosamine, this motif occurs in both N- and O- linked glycoproteins as well as in glycolipids. For example, in complex N-glycans, the core mannose can be decorated with up to five GlcNAc residues, each of which can potentially be extended into a linear chain of polylactosamine (Fig. 1*A*); which GlcNAc residues are extended varies by tissue and developmental stage. These extended “antennae” can be modified by branching off additional polylactosamine chains, by the addition of modifying groups including sulfate (*e.g.*, to form keratan) and fucose, and/or capped with sialic acids, or surface antigens (including ABO, HNK-1, or Lewis X antigens) (1). Polylactosamine is directly recognized by many lectins including galectins (2), while simultaneously serving as a scaffold for presenting critical cell surface markers; it therefore plays an important role in diverse cellular processes, including development, cell adherence, and immune function.

**Figure 1:**
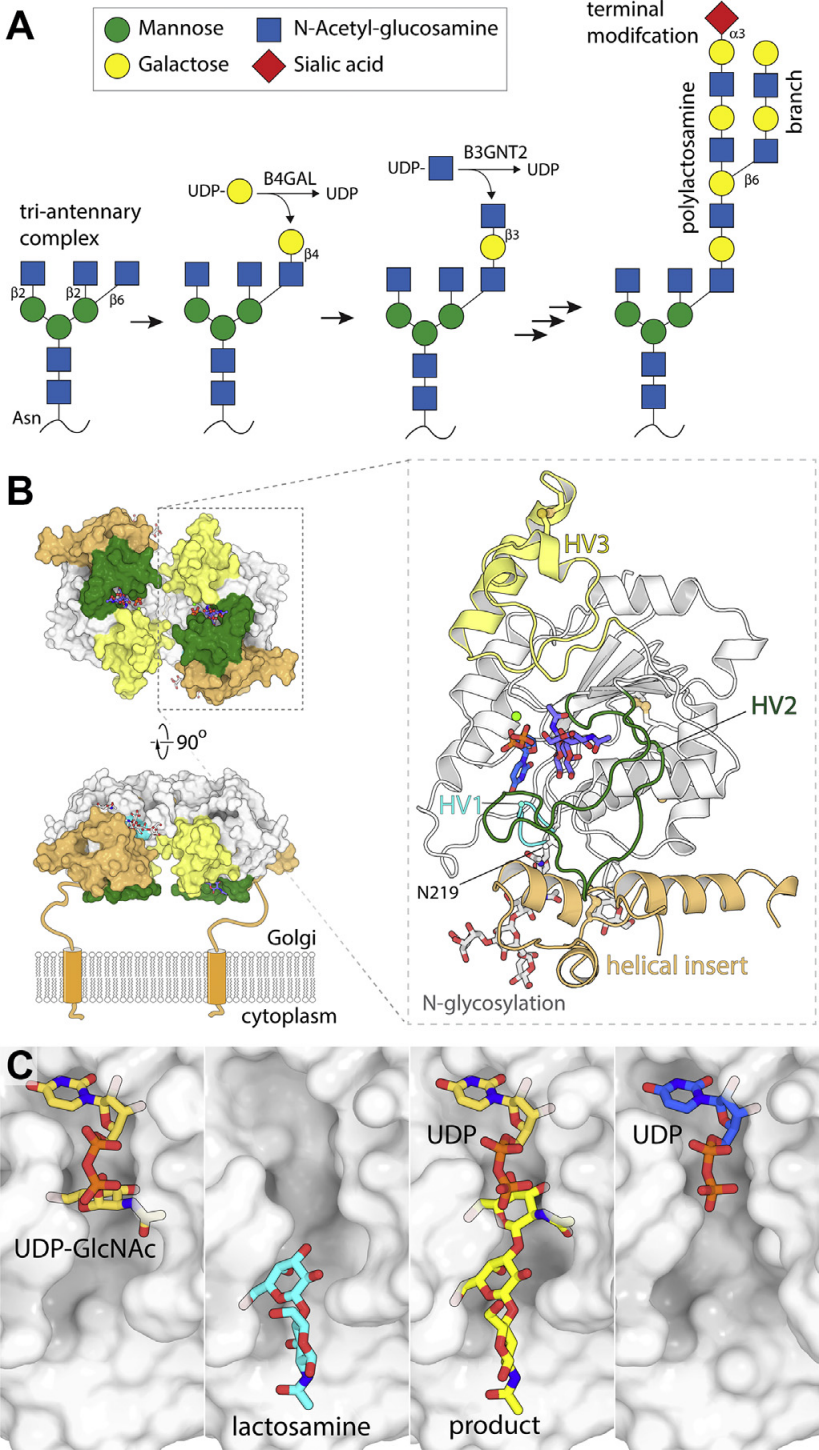
**The role and structure of B3GNT2.***A*, B3GNT and B4GAL collaborate to build polylactosamine antennae on N-glycans (shown) as well as O-glycans and glycolipids; these are then subjected to further modifications. *B*, a surface view of the UDP plus product complex structure of B3GNT2 (RCSB i.d.: 7JHN). The lower left panel shows a conceptual model of how this protein would be anchored to the Golgi membrane, while the right panel shows details of one protomer. *C*, key substrate and product complexes characterized in these works.

Glycans are synthesized by the sequential addition of monosaccharide units from nucleotide phosphate donors by enzymes known as glycosyltransferases (GTs). GTs occur in four distinct folds (namely GT-A through GT-D) and are further divided into homology-based families (currently, GT1–GT111). Individual GT families will transfer a specific monosaccharide with either inversion or retention at the anomeric carbon center (3). Polylactosamine synthesis requires the alternating action of two GTs: The Gal is added by β-1-4-galactosyltransferase (B4GAL), while the GlcNAc residue is added by β1-3-N-acetylgucosaminyltransferase (B3GNT). B3GNTs are classified as inverting GTs in family GT31 and are predicted to have a GT-A fold (4). In humans, B3GNT has seven isoforms termed B3GNT2–B3GNT8; these differ in their tissue distribution and substrate preferences, with many having specialized roles. B3GNT2 is a widely expressed, membrane-anchored, Golgi-resident enzyme that appears to be the most important homolog in elongating polylactosamine (5). To date, however, challenges of working with these enzymes have resulted in many key aspects of their biochemistry—including their structure, substrate recognition modes, and catalytic mechanism—being little understood.

In a pair of papers published in JBC, Hao *et al.* (6) and Kadirvelraj *et al.* (7) report the structure of the GT domain of B3GNT2, coupled with an in-depth analysis of the enzyme’s mechanism. Usefully, the groups used different approaches at several key junctures, so the papers provide nicely complementary insights. At the level of protein organization, the structures confirm a GT-A (single Rossmann domain) fold, but showed a number of B3GNT-specific modifications (Fig. 1*B*). Along with three disulfide bonds, the structure shows N-glycosylation at three sites, with the Asn219-anchored GlcNAc_2_ disaccharide packing in a cleft and likely critical for protein stability. B3GNT2 also has a unique N-terminal helical subdomain, as well as a long loop insertion (HV2) that forms most of the acceptor binding site. Finally, B3GNT2 is dimeric, while most GTs are monomeric. The active sites are on the same dimer face, but too widely separated to span different antennae on a single glycan. One might speculate that having paired, widely spaced membrane anchored tethers helps orient B3GNT2 optimally for substrate encounters.

GT reaction mechanisms can be difficult to study because labile nucleotide sugars and weak substrate binding can make capturing substrate complexes very challenging (3). Kadirvelraj *et al.* (7) cocrystallized B3GNT2 as UDP and as UDP plus trisaccharide acceptor complexes; they then modeled UDP-GlcNAc binding. Hao *et al.* (6) took a crystal soaking approach and were able obtain structures of an apo enzyme, the UDP–GlcNAc donor complex, as well as an acceptor complex and a product complex (Fig. 1*C*). Of particular interest is the acceptor binding site. In B3GNT2, only the two terminal saccharide residues make significant contacts with the protein, consistent with its activity being largely independent of substrate chain length (5). This binding site is built from extensions of three loops, with most contacts coming from a greatly elongated hypervariable loop 2. Using detailed comparisons to other GT-A structures, Kadirvelraj *et al.* (7) argue that GT-As generally evolve recognition to acceptors by extending and restructuring these hypervariable loops, allowing an enormous variety of acceptors to be recognized.

To complement these structures, Hao *et al.* (6) showed (using SPR) that both the donor and acceptor exhibit weak, fast-on fast-off binding. Combined with a detailed kinetics analysis, these data strongly suggest a sequential binding mechanism. Kadirvelraj *et al.* (7) also determined kinetics constants for both substrates for the wild-type and for ten variants in active site key residues, supporting the proposed mechanism. Hao *et al.* (6) assayed *in cell* function, assessing the ability of B3GNT2 active site residue variants to restore polylactosamine cellular expression. Together, these structures, mutagenesis, and enzymology experiments give a detailed window into the mode of substrate recognition and mechanism of this enzyme.

While these works are specifically focused on B3GNT2, GT31 is the largest family of GTs in humans (with 25 members) (4), and only the UDP complex structure of one other member is known. These papers therefore provide a “Rosetta stone” for understanding multiple enzymes with a variety of functions. Most immediately, there are six additional B3GNTs with varied biological roles and distinct acceptor substrate specificity. For example, B3GNT5 only synthesizes lactotriaosylceramide (5), while B3GNT7 synthesizes keratan and strongly prefers sulfated substrates (8). These differences imply extended and/or modified paralog-specific recognition sites. Modeling based on these structures will allow key motifs and residues mediating such specificity to be identified. Finally, B3GNT2 pairs with B3GNT8 to form heterodimers that are more active than either enzyme alone (9); the structural and enzymatic basis of this synergy represents an intriguing subject for future investigations.

## Conflict of interest

The author declares that he has no conflicts of interest with the contents of this article.
